# The Drawbacks of Project Funding for Epistemic Innovation: Comparing Institutional Affordances and Constraints of Different Types of Research Funding

**DOI:** 10.1007/s11024-017-9338-9

**Published:** 2018-01-09

**Authors:** Thomas Franssen, Wout Scholten, Laurens K. Hessels, Sarah de Rijcke

**Affiliations:** 1 Center for Science and Technology Studies (CWTS), Leiden University, PO Box 905, 2300 AX Leiden, The Netherlands; 2 Rathenau Institute, Anna van Saksenlaan 51, 2593 HW The Hague, The Netherlands; 3 KWR Watercycle Research Institute, PO Box 1072, 3430 BB Nieuwegein, The Netherlands

**Keywords:** Research funding, Projectification, Prize, Epistemic properties of research, Competitive funding

## Abstract

Over the past decades, science funding shows a shift from recurrent block funding towards project funding mechanisms. However, our knowledge of how project funding arrangements influence the organizational and epistemic properties of research is limited. To study this relation, a bridge between science policy studies and science studies is necessary. Recent studies have analyzed the relation between the affordances and constraints of project grants and the epistemic properties of research. However, the potentially very different affordances and constraints of funding arrangements such as awards, prizes and fellowships, have not yet been taken into account. Drawing on eight case studies of funding arrangements in high performing Dutch research groups, this study compares the institutional affordances and constraints of prizes with those of project grants and their effects on organizational and epistemic properties of research. We argue that the prize case studies diverge from project-funded research in three ways: 1) a more flexible use, and adaptation of use, of funds during the research process compared to project grants; 2) investments in the larger organization which have effects beyond the research project itself; and 3), closely related, greater deviation from epistemic and organizational standards. The increasing dominance of project funding arrangements in Western science systems is therefore argued to be problematic in light of epistemic and organizational innovation. Funding arrangements that offer funding without scholars having to submit a project-proposal remain crucial to support researchers and research groups to deviate from epistemic and organizational standards.

## Introduction

 Over the past decades, science funding shows a shift from recurrent block funding towards project funding mechanisms (Hicks 2012). This shift takes place against the background of an economization of the public sphere, a rise of ‘technologies of government’ (Miller and Rose 1990) and of audit processes in a wide variety of sectors, including higher education (Dahler-Larsen 2011; De Rijcke et al. 2016). Increasingly, new funding instruments aim at differentiating between ‘normal’ and ‘excellent’ science (Aksnes et al. 2012). Selective support to the best scientists would improve the overall quality of science as well as drive ‘average’ scientists towards better achievements (in their attempts to be recognized as excellent). As a result, yet at different speeds in different scientific domains and different public science systems, we see an increase of project funding arrangements in Europe from the 1980s onwards (Lepori et al. 2007). However, our knowledge of how project funding arrangements influence the organizational and epistemic properties of research is limited.

Scholarly attention to the effects of new funding arrangements has been most prominent in the field of science policy studies where scholars have discussed the impact of the shift towards project funding on the macro level. There is relatively little empirical work on the relation between funding arrangements and organizational and epistemic properties of research (but see Heinze 2008; Heinze et al. 2009). To study this relation a bridge between science policy studies, strong in the analysis of funding mechanisms, and science studies, strong in the analysis of organizational and epistemic properties of research, is necessary (Gläser and Laudel 2016). In recent years, in relation to the rise of the ERC funding schemes, a group of studies that aim to do exactly this has appeared. These studies analyze the relation between the affordances and constraints of project grants and the epistemic properties of research (Laudel and Gläser 2014) as well as organizational changes, for instance in authority relations (Edler et al. 2014; Gläser et al. 2010; Whitley 2014).

However, the potentially very different affordances and constraints of funding arrangements such as awards, prizes and fellowships, have not yet been taken into account. The available studies on the relationships between funding arrangements and epistemic properties deal exclusively with project grants. Drawing on the analytical model developed by Laudel and Gläser (2014) and eight case studies of prize and project funded research, this study aims to compare the institutional affordances and constraints of prizes, in this case the Dutch Spinoza prize, with those of project grants, in this case the Dutch Vici grant and the European ERC Advanced Grant, and the effect they have on organizational and epistemic properties of research. In order to sharpen our analysis, we focus on highly prestigious project grants that are more similar to prizes than most available project grants. While most project grants are awarded using a combination of thematic criteria and academic merit, the grants we analyze are based on academic merits only. What is more, they offer a large degree of autonomy to the researcher about how to spend the money. The main difference between the two funding arrangements studied here is the existence of a project proposal (including a peer-review procedure organized around this proposal) in project funding arrangements. Prizes also have peer-review processes but these are to judge past performance. This methodological focus on extreme cases of project grants allows us to extrapolate our findings to many other types of grants, because most other project grants will differ more from prizes than the ones studied here.

A comparison between project and prize funded research in the same research groups (and partly of the same researchers) offers the analytical opportunity to assess the effects of such a selection procedure. Prizes are awarded without the researcher having to make explicit how the funding will be used. We ask what kind of institutional affordances and constraints do prizes and project grants offer, and how do these characteristics influence the organizational and epistemic properties of academic research?

We argue that the prize case studies diverge from project-funded research in three ways: 1) a more flexible use, and adaptation of use, of funds during the research process compared to project grants; 2) investments in the larger organization which have effects beyond the research project itself; and 3), closely related, greater deviation from epistemic and organizational standards. The two latter elements show the intertwined nature of the organization of an institute, group or research project and the epistemic properties of research done in an institute, group or research project. We argue that distribution of resources through project grants epistemically constrains even the winners of project grants. The increasing dominance of project funding arrangements in Western science systems is therefore argued to be problematic in light of epistemic innovation.

## Analytical Framework

Important advances have been made to connect the epistemic dimension of scientific practice, i.e., how scientists produce knowledge, to the science systems in which these practices take place. While these two domains are often studied separately in the fields of science studies and science policy studies (Gläser and Laudel 2016), recently scholars have begun to connect these fields empirically and theoretically and have developed models that aim to understand the relation between funding arrangements and epistemic and organizational properties of research.

### Project Grants

Research in science policy has documented the shift from recurrent block funding for universities, that can be used to allow for a minimum amount of research time for all staff members, towards project funding arrangements as the most important development of the last three decades (Lepori et al. 2007). The rise of different types of grants has important effects on the organization of research. Scholars have shown how the rise of prestigious large project grants in the European Research Area influence authority relations between university managements and research group leaders (Edler et al. 2014) and how research practices are shaped in new interdisciplinary organizational constellations through the increase of grant size (Bloch and Sørensen 2015). Laudel and Gläser (2014) offer an analytical model of epistemic properties of research funded through project grants and its institutional affordances and constraints (which will be explained in more detail below).

A minimal definition of the project funding arrangement is that participants are obliged to write a project proposal around which the competitive selection procedure is organized. In our case, to ensure comparability with prizes, we have selected project funding arrangements in which selection is based on the academic merit of the applicant and the proposal (rather than thematic project grants).^1^

### Prizes

The scientific prize is an important medium of recognition in the reward system of science (Zuckerman 1992). Already in 1992, Zuckerman observed a proliferation of prizes in the US, with increasingly large sums of money attached to them. Zuckerman argues that it was the Nobel Prize and its scarcity and accompanied prestige that has led to the proliferation of prizes in the sciences, especially in fields outside the four fields in which a Nobel Prize is awarded. According to Zuckerman, the effect of this proliferation of prizes on the reward system is limited; the number of prizewinners remains small and often prizewinners go on to win other prizes as well, making the number of individual prizewinners even smaller.

It is, however, time to reconsider this. Zuckerman notes that next to the proliferation of prizes that are honorary in nature, ‘an additional half-dozen new awards are equally lavish but differ from the purely honorific awards in that they are designed to provide support for future research’ (Zuckerman 1992: 218). These prizes, premiums or fellowships such as the Louis-Jeantet Prize for Medicine and the MacArthur Fellow Awards consist of relatively large sums of money (these two both over half a million Euros) and are aimed to push the research agenda of its grantees forward. It is the proliferation of this type of prizes that plays an increasingly important role in the science system. The minimal definition of prize funding arrangements is that they are awarded by a selection committee without the grantee having to, beforehand, make explicit how (s)he would use the funding. In this case, we have selected a prize which is seen as the most prestigious in the Dutch science system specifically aimed at scholars in the most advanced career stage.^2^

### The Analytical Model

**Fig. 1:**
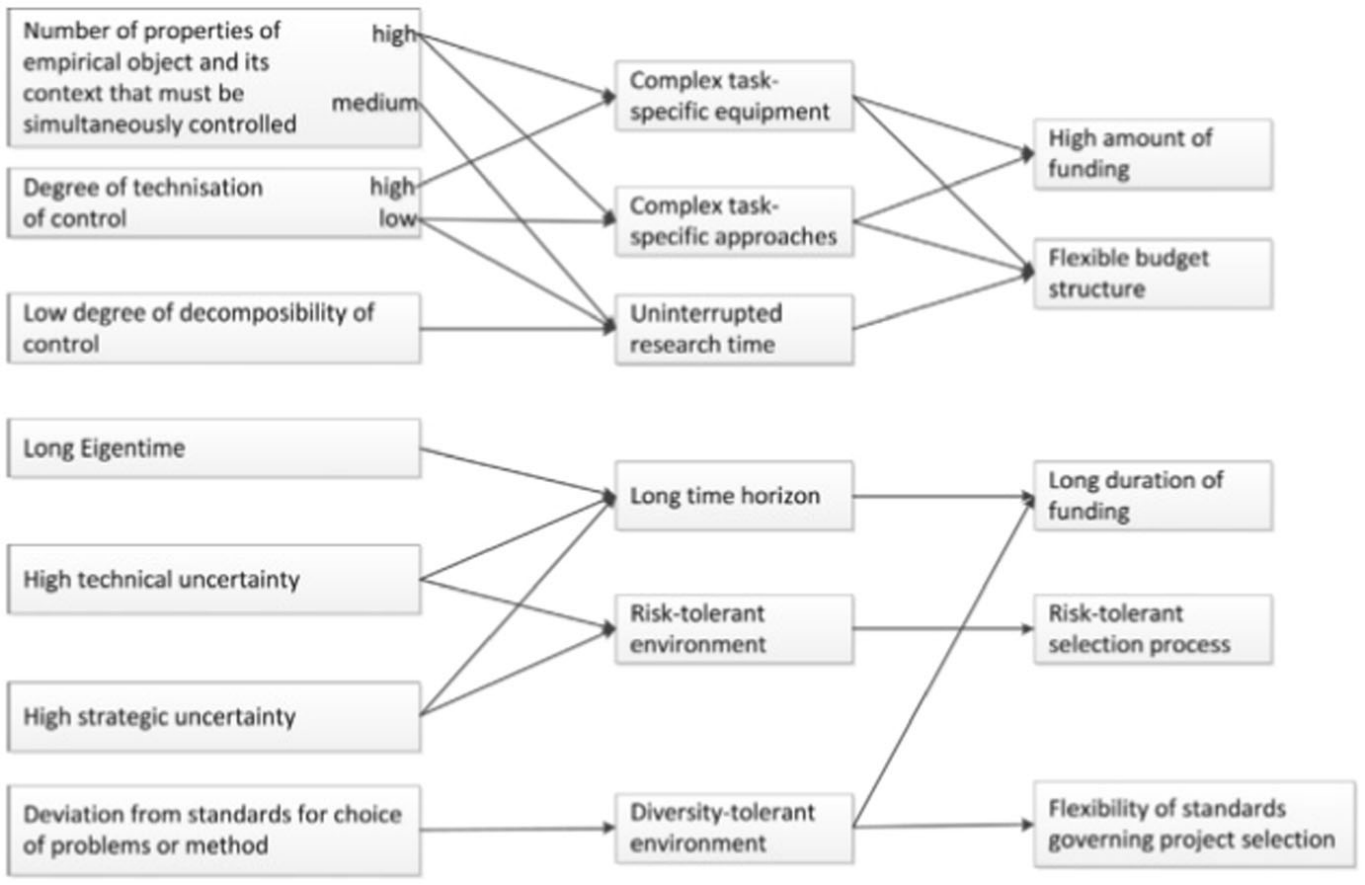
Analytical model of relation between institutional affordances and constraints and epistemic properties (Reproduced with permission from Laudel and Gläser 2014: 1211)

Laudel and Gläser (2014) analyze the epistemic properties of research funded through ERC Starting and Advanced Grants. They connect these properties, via necessary and favorable conditions for research, to the institutional affordances and constraints of ERC funding arrangements (see Fig. 1 for their analytical model).

The institutional affordances and constraints identified in this model can be captured in two categories. First, the selection process. This process can be more or less risk tolerant and can allow for a greater or lesser flexibility in (epistemic) traditions that are deemed acceptable and fundable. Second, the characteristics of the funding arrangement itself, its duration, the amount and the extent to which the budget structure is flexible.

Regarding our comparison of project grants with prizes, we expect important differences in these institutional characteristics. First, prizes have a different selection process. Importantly, there is no project proposal that is peer reviewed by external reviewers or a peer review panel. This is important as peer review panels tend to be relatively conservative in their judgment of project proposals (Luukonen 2012), especially in funding programs that are characterized by a high level of competition (Langfeldt 2001). Second, the lack of a project proposal also implies there is greater flexibility after the prize is granted in how money can be spent. Project grant holders are incentivized to follow their project proposal, for instance, the organizational aspects (such as the research team composition), and the theme of the proposal.

We expect that the absence of a project proposal has important organizational and epistemic implications. Following Laudel and Gläser, we distinguish four core epistemic characteristics of a research project. First, the relation between the researcher and the epistemic object. This includes the number of properties of an epistemic object that the research has to ‘control’ to produce data about the object, and the ‘technisation’ of control, as well as the extent to which control can be divided across different researchers and different sites (decomposability of control). For instance, a Bose-Einstein condensate of cold atom gases can only be achieved by combining three techniques and keeping the gases in a state of an extremely low temperature and an extremely high vacuum (Gläser and Laudel 2015: 318). In such a case, the number of properties that have to be controlled is high and so is the technisation of control. The decomposability of control is low because this experimental setup has to be created in one laboratory and cannot be divided across different sites.

The second characteristic is the eigentime of the epistemic object. This is the time which the research process necessarily takes. This varies, for instance, because of the sequence of steps that has to be taken to produce data or because of the breeding cycles of model organisms (Gläser and Laudel 2015: 316, 323).

The third characteristic is the strategic and technical uncertainty of the research. Strategic uncertainty is the uncertainty regarding the question whether the effect that is being sought actually exists. The technical uncertainty is the uncertainty whether the effect or mechanism can be uncovered using the method of choice and whether researchers are able to successfully implement such a method (if it depends on a highly novel or complex experimental setup, for instance) (Gläser and Laudel 2015: 316). Taking the Bose-Einstein Condensation as an example, this was for a long time very uncertain both strategically and technically. For each new type of atom it was not clear whether the desired effect would arise while technically the combination of multiple methods created a high technical uncertainty (Gläser and Laudel 2015: 320).

The fourth characteristic is the extent to which researchers deviate from the standards in their research tradition. We understand this deviation to include both organizational and epistemic properties of research, the former would include the organization of research and publication practices while the latter would include method, problem, epistemic object or scope.

The difference between institutional characteristics of prize funding in comparison with project grants is expected to alter organizational and epistemic properties of research especially in the latter two characteristics. We argue that the most important difference is the lack of a project proposal in the selection procedure, this leads us to hypothesize that prize funded research will be organizationally and epistemically more diverse as prizes enable researchers to deviate from epistemic and organizational standards. They might take on new problems, new methods or new epistemic objects and organize their research in a novel way.

## Data and Methodology

The current study is part of a larger research project on the rise of competitive funding (Hessels et al. 2016). We carried out four in-depth case studies of research groups who obtained several project grants and prizes to analyze the role of competitive funding in the organization of research in high performing research groups. During the analysis the present focus on the differences between the affordances and constraints of project grants and prizes emerged. From the four high performing groups we selected eight research projects funded by prizes or project grants for the present study.

To come to the initial selection of four high performing groups we used several criteria. For the groups’ performance we looked at their scores in the periodic research reviews and important prizes and project grants won by group members. To select groups in a variety of research cultures that deal with different epistemic objects we followed Whitley’s (2000) differentiation of ‘the degree of strategic task uncertainty,’ and added the level of collaboration as a second dimension (Tsai et al. 2016). We concluded that selecting research groups from the humanities, the social sciences, mathematics and geosciences, would afford variation on these variables.

In the humanities we selected a research that studies cultural history of different cultural communities in a specific historical period. Our social science group is positioned on the borders of two social scientific disciplines where it studies aspects of the life course. The subfield of the mathematics group can be defined as number theory with both an interest in theory and application. The geoscientists also work in an interdisciplinary research area which combines chemistry, biology and geology to, among others, study the climate.

Over the past 15 years researchers in the four groups have received funding from a diverse range of funding arrangements, such as governmental recurrent block funding which includes a research budget that is commonly used to fund research time for permanent or tenure track staff members, project grants from various sources and prizes. To compare project grants with prizes we focus on those awarded to senior researchers. We analyze the usage of the Spinoza prize that was received by three researchers in three of the groups. We compare the Spinoza prize to the usage of the ERC Advanced Grant (received by three researchers in two groups, partly of which one also received a Spinoza prize) and the Vici grant (received by two researchers in two groups without overlap with the Spinoza prize) (Table 1). We chose to focus on these project grants and prizes because they are all awarded primarily based on academic merit, are prestigious, large in size and less risk-averse compared to other project grants as these project funding schemes have no epistemic or organizational limitations as to what researchers can propose. These characteristics make these types of project grants more similar to academic prizes than most other project grants.

**Table 1:** Overview of cases

Funding type	Discipline	Amount
ERC Advanced Grant	Social sciences	2.5 million
ERC Advanced Grant	Geosciences	2.5 million
ERC Advanced Grant	Geosciences	2.9 million
Vici grant	Geosciences	1.25 million
Vici grant	Mathematics	1.25 million
Spinoza prize	Geosciences	1.5 million
Spinoza prize	Mathematics	1.5 million
Spinoza prize	Humanities	1.5 million

In order to protect our respondents, this paper will not reveal the particular groups that we have studied. For the same reason we also conceal the gender of respondents and refer to all of them as female.

For the larger research project we interviewed between 9 and 11 group members of all career levels and in some cases an organizational superior (like a dean or head of department) or an informed outsider (like a department professor or review committee member). The recipients of these eight grants and prizes were all among the respondents and in two cases they were interviewed twice. In all interviews with senior researchers the use of, and differences between funding sources were discussed. We complemented our data collection by doing a document analysis of the submitted project proposals and formal or informal evaluations written by researchers for funding bodies. The data were categorized using the interview topics as the initial coding categories (supported by Atlas.ti). We then extracted all interview segments which talked about the properties of prizes and project grants and their relation to the research projects. The majority of examples discussed in the article come from the interviews with the recipients but we support our analysis with quotes from group members on other career levels who are involved in the research.

## Analysis

### Institutional Affordances and Constraints of Project Grants and Prizes

The two project grant funding arrangements we consider in this article are the ERC Advanced Grant and the Vici grant. The ERC Advanced Grant consists of 2.5 million Euros over a 5-year period and is seen as highly prestigious. The Vici grant is part of the Innovational Research Incentives Scheme, a funding program developed by The Netherlands Organisation for Scientific Research (NWO), aimed at high performing individual researchers. The Vici amounts to 1.25 million Euros to cover 5 years of research. The selection process of both is similar and uses a project proposal that is reviewed by external reviewers and a peer review panel (see Luukonen 2012 about the ERC).

The prize we consider is the Spinoza prize. Instigated in 1995, the prize started at approximately 1 million Euros (fl. 2 million) but in 2009 it increased to 2.5 million Euros. The prize is awarded yearly to a variable number of scholars (between 2 and 4). The duration is officially five years, but can be extended (according to one of our interviewees up until official retirement). Researchers can not apply for the Spinoza prize; they can only be nominated by others. Moreover, nomination can only be done by a select group of people such as the principals of Dutch universities and chairs of the Royal Netherlands Academy of Arts and Sciences. The selection is done by a peer review committee, using external reviews and other information (such as bibliometric analysis) to decide who is awarded with the prize.

### Organizational and Epistemic Properties of Research

In this section we discuss the research projects funded through the two funding arrangements discussed above. We describe the organization of the research process to gain insight in the budget structure and planning of the project, as well as in epistemic properties that are relevant for the comparison.

### Epistemic and Organizational Characteristics of Project Funded Research

The social science ERC Advanced Grant deals with a research object at the interface of two different areas of social science. By connecting these two areas and addressing various limitations of existing literature, the project aims to increase the understanding of this complex phenomenon. The project contains four sub-projects, which each address a distinct question and in this way each fill a particular gap in the existing literature, such as gender aspects and the relative influence of national policy contexts.

The project generates a substantial body of new data by a large-scale novel survey on a specific population. The researchers argue that this particular data collection is so laborious that it would have been difficult to fund it without the ERC grant. One postdoc researcher has worked for two years on data collection, without having time for any data analyses. Beside these new data, the project analyzes various types of existing data. In this sense it builds on existing data infrastructures. The project is in fact only possible thanks to the availability of advanced datasets in various countries.

The project is carried out by a core team of two PhD-students, one postdoc researcher, one senior researcher and the project leader (full professor). This core team is embedded in a larger team of affiliated researchers working on similar topics. For example, one PhD-student receives additional supervision from an associate professor not formally tied to the project.

The project addresses a large number of properties of the object of study. The control over these properties is achieved through a rigorous survey methodology, which builds on prior experience of the project leader in large-scale data collection. Although the project uses advanced statistical approaches, the analyses can be carried out on regular computers. This form of technization of control offers good possibilities for decomposability of control. Each researcher is responsible for a certain subproject and makes a distinct contribution to the project as a whole. The interlinkages among the researchers’ activities is, however, limited. This can be illustrated by the perception of a group member that there is no clear distinction between researchers working within the project and others.

I don’t see a difference. Because I think the thing is that even though [colleague’s name] is on a different grant, she is under [professor’s] sort of umbrella and the [theme] group. So, I mean, that is also something that you see, is like the lines between who is on what grant, are really blurred. (PhD-student, social science, 2015)

The technical uncertainty of this project is relatively low as the data collection strategy builds upon prior data collection initiatives of the project leader and well established survey methodologies. The project makes a substantial investment in data collection, carrying out a large-scale survey among a specific population that is not easy to reach which creates uncertainty whether sufficient response will be achieved. However, the project also works to a great deal with existing datasets. If all research tasks are carried out as planned, the project will certainly yield results. What remains uncertain, however, is the degree to which statistical relationships will be found, which we understand as part of the strategic uncertainty. One subproject suffered from this issue. The PhD-student made an ambitious cross-national comparison, but did not find any statistically significant correlations. This made it difficult to publish the results as the hoped for effect was not found. During the execution of the research, the researchers have a certain flexibility in the degree of strategic uncertainty they accept:

You know, it is also a bit with these publication strategies, at the moment I happen to have some ideas that are more difficult to carry out and one that is simply a safer choice, and I had to prepare a workshop and I thought like I just have insufficient time to start doing complicated things, then I find it too risky and that I just don’t think is alright, because in the end I might just have nothing to present, so I simply go for these safer ideas. (Postdoctoral researcher, social science, 2015)

The project is innovative in its intellectual scope and combination of various subdisciplines, and for the use of state-of-the art data and statistical methods (such as Bayesian statistics). However, it is not controversial in the sense that it strongly deviates from epistemic standards of the field. Its activities build on generally accepted bodies of literature and robust datasets and ways of data collection.

In the geosciences group, three project grants were obtained which could be included in our study. The group leader received an ERC Advanced Grant and the second professor obtained a Vici grant and an ERC Advanced Grant. Interestingly, the epistemic structure of all three research projects is very similar. The research group works on reconstructing the earth’s climate and its evolution using sediment samples. In these samples, originating as far back as the Mesozoic era, (fossilized) organic sediments are found. The properties of these organisms can be used as a proxy for external conditions such as temperature, thereby creating what is called a paleothermometer. In all three research projects the researchers aim to further develop different recently discovered proxies. They write:

Recently, my group discovered a new [technical detail] proxy, the [name of proxy], which is based on the [aspect of sediment]. Their composition is a function [technical detail]. (…) This project (…) will bring this novel [technical] paleothermometer to maturity. (ERC Advanced Grant proposal)

The [name proxy] has been developed in my group and has already shown to yield unique and unprecedented information on past [technical detail proxy]. (…) The [name proxy] is a unique proxy which was only developed this year. If the initial results with this index are confirmed, then it would give an unprecedented tool to reconstruct [technical detail proxy] over long, geological time scales. (Vici proposal)

The overarching objective of this ERC project is to develop [name proxy] as a proxy for [technical detail of the proxy]. (…) Initial results discussed above have clearly shown the high potential of [name proxy] as novel [technical detail of proxy] proxies. In contrast, the development of the [name proxy] as a [technical detail] proxy is clearly ‘high risk, high gain’ research as only limited pilot data has been acquired yet. The mix of medium- to high risk research with the number of high gain results targeted, makes this project ideal for an ERC application. (ERC Advanced Grant proposal)

In all three projects recently discovered proxies were further developed. Specific parts of the ERC projects were deemed high risk by the researchers while the majority of the projects were less uncertain. It was already clear that the organisms, their epistemic objects, were proxies and the research consisted of developing them further to understand what they could and could not be used for. In an interview, the second professor explains that the real ‘breakthrough moment’ was the moment the group discovered that a particular organism or property of an organism could be used as a proxy:

My ERC Advanced Grant is exactly the same. That proposal is based on an accidental finding by [name postdoc]. She discovered certain patterns in the data that we were collecting for completely different purposes. This resulted in a very nice paper and I took that work as a basis, with other things of course, for the ERC proposal. But there was a basis and the innovative breakthrough had already happened. This now comes down to developing it and that also is time-consuming and costly. (Professor, geosciences, 2015)

The three projects were also organized in a similar way, employing a mix of PhD-students, postdocs and technicians. The postdocs were employed on the more high-risk parts of the projects while the PhD-students would do work that is time-consuming but lower in risk (as they had to come out of the project with a completed dissertation). The organizational model resembles to a high degree the ideal-typical lab-structure that we know from the life sciences (Conti and Liu 2015). This organizational model is characterized by pyramid-like organizational structure with a PI at the head of a group of PhD-students and early-career researchers. Scientific work is in practice carried out by PhD-students and postdocs, but analyzed and published in collaboration with the project leader and other senior members of the lab.

The epistemic properties of the three projects can be described in similar terms. The amount of properties that have to be controlled is high, as is the technization of control which includes the need for task-specific, high-quality equipment. The decomposability of control is high. The projects are divided into PhD and postdoc projects, and in each project different senior researchers are assigned to assist with their expertise. Moreover, from the evaluation of the Vici project it becomes clear that the number of publications that originated from the project is very large (>100), which shows that the knowledge claims that are publishable are smaller or more rapidly accumulated than in other fields. However, we observe that these publications operate at different analytical levels. Some are more ambitious in their scope than others and, for instance, bring together data from a large variety of sediments, while others are case-studies of particular locations. The strategic and technical uncertainty of the projects is relatively limited because the relationships between the properties of the epistemic object and the external conditions - making them suitable as proxies - have already been discovered. However, there is uncertainty about the extent to which they are good proxies, the number of factors for which they act as proxies and their overall limitations as proxies (because of systematic variation, and alternative causes for changes in their properties). The novelty of the research project lies in the development of new proxies that are not being used in the field. But again this is limited as in prior research the group has already established the use of these epistemic objects as proxies.

In the mathematics case study, the Vici project aims to quickly and efficiently count solutions of systems of a specific kind of equations, by way of smart novel algorithms. The strategy for this project was inspired by a colleague who stated that the solution of the problem, already posed in 1995 by another colleague, might not lie in using computer algebra, but in using numerical analysis. It was necessary to estimate a required precision of numerical computations and the novelty of this project was to make these numerical approximations by using numerical analysis. What made the project stand out was how insights and methods from other mathematic subfields, numerical analysis, computer algebra and number theory, were brought into arithmetic geometry as a novel strategy to answer the research question.

The importance and novelty of the research project were recognized by peer reviewers as the most important current work within the subfield. The researcher documented the trajectory of her Vici project, from proposal up until the outcomes in an article. There she cites the review she received saying that the reviewer expects the researchers to achieve at least part of their goals and that it will be an important landmark study in the subfield.

However, in the application as well as the interview the professor is careful in explaining she sees the project not as inherently or overly risky. In mathematics in general strategic uncertainty revolves around the question whether a certain problem can be solved or whether a conjecture can be proven. If one knows it can be, then the most uncertain part of the research process is already completed, but it is hardly a challenging or worthwhile endeavor to write a project proposal about such a problem. After all, the solution or proof is already guaranteed and peer reviewers will know so.

The strategy of the professor in this project was to use numerical analysis for the problem that until then was approached by computer algebra. Over the course of several years, in which she worked on the problem and also submitted a (rejected) Vici proposal, the professor was able to minimize the risk that there would be no outcome. She found some evidence that the proposed strategy to work on a solution would succeed. What had been a highly complex and high risk problem ten years before submitting the proposal had been reduced to a manageable research project a few years later.

That was 2000 [when the proposal was rejected], and then I worked out a strategy but the project was on hold for two years. So two years later I started to work on the project again as I had thought it through. I could show that most probably it would all work like I thought it would. So the second time I had already worked on it for a year because I did not put it on hold, and I had a lot more… indications that it would work.

*Interviewer:* So less risk of failure?

Yes, I don’t know why people want risk in research. High risk, high gain they say, but risk… I would not say that is something positive. I would say, if you have high risk you should… compensate that… why didn’t you try harder to show that it will work? Or why didn’t you find out beforehand whether it would work?

*Interviewer*: Is that typical for mathematics?

It is of course very different if you study… if you ask a lot of people how they think about something, I mean, that is really different. In mathematics you have to construct the proof, you must have a theorem for which you want to give proof. That is just your work and not the result of a measurement or something, it is just proof. You must construct it and others will check if it is correct. (Professor, mathematics, 2015)

Because mathematics does not rest on empirical validation or a data-driven approach, the relation with risk and uncertainty is different from the other fields in our case studies. The quote from the interview suggests that the professor does not accept uncertainty to the extent that she envisions it to be present in other fields (such as the social sciences to which she refers). In this particular epistemic culture, the work has to be done by the individual and without input from empirical data. Therefore, there is a need for a high degree of clarity on the way in which a problem will be approached and for indications that such an approach will work.

While the professor claims that mathematics consists of highly solistic work, the Vici project contained three postdoc positions (two years each) and three PhD positions (four years). Some of the individual projects that these junior scholars would carry out are already identified in the application, and are often adjacent to the main problem the professor aims to solve. This shows that while the work mathematicians do is solitary, in project proposals the research is fitted to the project-form and well-known forms of lab-like collaboration.

### Epistemic and Organizational Characteristics of Prize Funded Research

The research area of the professor in the humanities group spans a long period and the entire European continent. The focus on this particular period is not uncommon, but the comparative perspective on different regions within Europe was relatively new when the scholar began her career at the end of the 1980s. The research project, funded by the Spinoza prize and a second equally prestigious prize, revolved around an ongoing concern regarding the rise of a certain phenomenon in different cultural communities in Europe. In an article published a few years before she received the two prizes, the professor already envisioned what the field needed to progress:

That was a fundamental article. (…) In it I looked at ways to operationalize ‘culture’ (…) I have sorted it and I have mapped fields and media, we have reflection about language, things done with texts (…) we have material culture, that are buildings, archeology, dolmens, and so on. We have immaterial culture, music, dance, and if you sort that, then you get an ending number of fields of which you can say ‘this is happening.’ (…) That was before I received the Spinoza prize. Then I said, an encyclopedic full inventory of all these things would be impossible. Then I received 1.5 million and I said, it is possible and that became the encyclopedia. (Professor, humanities, 2015)

Her ambition was thus to bring together knowledge about all forms of culture from all main cultural communities in Europe over a long period, but she could not do so alone. She had to bring together around 500 scholars to contribute their expertise. A group of student-assistants collects data directed by the professor or the outside experts. Moreover, the contextualization of the data happens through an encyclopedia filled with articles from the experts in this network.

These are the people with whom we work. They do different things. We invite them repeatedly for workshops on different scientific themes. (…) All data in the database is brought together by 500 people that we give a weekend in [university town] and a workshop and the student-assistants. It will all come together in the encyclopedia that in this way is carried by 500 ‘employees’ from 40 countries and that is ran by six student-assistants. That encyclopedia will be [describes theoretical intervention in the debate] forever. (Professor, humanities, 2015)

The number of properties that have to be controlled is very high, both the spatial and temporal scope of this project are unique within the discipline. The decomposability of control is variable. On the lowest level, that of data collection and description of particular cultural expressions, there is a large decomposability. Student-assistants and foreign experts can all do part of the tasks at hand. However, on a higher level of abstraction, that of theorizing across these cultural communities, the decomposability is much lower as the professor has to combine this knowledge to come to a theoretical synthesis. However, while the professor has to develop her theory individually, other scholars can work with the data gathered in this project. There is some technization in the project; a database was developed by a small creative company who also developed ways of visualizing and presenting the data online. This technization is not aimed at an analysis of the data in a quantitative manner but to make the data and results available for the scholarly community.

The strategic uncertainty of the project is relatively low. The central hypothesis propagated by the professor that the same cultural phenomenon has taken place across different cultural communities in Europe within a particular period was already developed by her in previous publications. It is established in the field as a contender with a socio-economic structuralist explanation for the same phenomenon and has a strong presence in the scholarly debate. The theory assumes that in each cultural community the phenomena that the researchers investigate has become visible in material or immaterial cultural expressions. The technical uncertainty of the project lies in the novelty of the method and scale of data collection and analysis, of all these cultural expressions, as much of the data needs to be collected specifically for this project. The researchers cannot draw on existing national or international databases.

The deviations of existing practices in the research tradition in which this professor works are on the novelty of the method, the size of the research object and the organization of collaboration. In contrast to common practice, this project does not build on PhD-students, but on student-assistants, a few partly involved staff members, and a large network of scholars that collaborate for a smaller or larger part of the project. Collaboration is primarily organized on the level of data collection and a first step of data analysis (of individual cases), while the synergizing work is done by the professor, and other scholars, in a more individual way and without a research group consisting which, she argues, is not the most effective way to do research in the humanities.

I did not have straightforwardly successful results [in a funded project with a project grant] with regards to the productivity of group work and PhD-students working on command in the humanities. (…) I do not feel the need to create and institutionalize a group. I have seen that when a professor leaves you do not know whether it will continue to exist. I find it much more important that my method gains recognition. My method is very specific. I have the ambition to radically change [research theme], as it was once radically changed by [famous scientist], I want it to be totally changed after [own name]. That is coming. That is the big ambition. (Professor, humanities, 2015)

She later explains that the extent to which you can scale up in this type of humanities research is limited, but her research practice shows this limitation is primarily within the more analytical theory-driven part of the work rather than the level of data-collection and individual case studies. Rather, we would argue, collaboration and scaling up have a different form than in traditional natural science research. In the latter, collaboration is part of each phase of the research project, and recognition in the form of authorship is assigned to all scholars who contribute to different phases.

In the research project of the professor, data collection and data analysis (including workshops in which scholars get together to discuss results) are collaborative phases of the research process. Scholars contribute to the project in many different ways, but recognition for this is not translated into authorship on particular collaborative publications. Rather, in this epistemic culture scholars are expected to develop themselves as independent scholars and single-authored publications are an important part of this. This is, in this project, made possible through the encyclopedia for which many experts contribute a single-authored chapter and which is edited by the professor and a postdoc.

The second Spinoza prize in our case study material, in the geosciences group, was used in a different way. The group leader did not spend the prize on a particular project but, initially, on the hiring of a new tenure track staff member. This decision was as much epistemic as it was organizational. The primary epistemic object of this group are organisms that are uncovered from sediment samples and their microbiological makeup. A senior researcher explains the area on which the group works:

[W]e have lipids, these molecules that are surrounding a cell, (…) because as they are surrounding the cell, they are in permanent contact with the outside. So, if there is any stress, the lipids are going to change to be able to face the stress. (…) We use the lipids as a tool to reconstruct temperature in the past. But of course because these lipids, as I was saying, they are sometimes found in very specific microbes, they can be also used as tool for recognizing the presence of those microbes. (Senior researcher, geosciences, 2015)

Using lipids as a tool enables the group to do all kinds of comparisons of external conditions across time and space. This field has always been interdisciplinary, and the group works on the borders of chemistry, biology and geology. The new tenure track researcher was supposed to add a new expertise to the group, genetics. The group leader explains:

At a certain time I noticed that we need to do a lot more in molecular genetics. So then I decided, ‘we need a new tenure tracker in that area.’ So then of course I tried to convince the management that we should hire someone like that. (…) They didn’t go for it. So after a while I decided to use my Spinoza prize to hire a tenure tracker. I agreed with the management, ‘Ok, I will pay the salary for the first five years but then you have to take over.’ (…) But that was a typical example of, ‘ok, we need more knowledge in this area, so we really need to bring someone in that can bring this.’ (Professor, geosciences, 2015)

The new tenure track researcher had prior knowledge of the research area but had to adapt, as using genetics in service of another discipline entailed a shift in focus. In the years after the new researcher started working in the group, the collective had been able to advance the molecular genetic techniques it uses and is now on a new frontier, trying to explain the source of lipids by means of molecular genetic work. She explains:

When I started, we were doing really basic molecular genetic techniques to be able to answer various specific questions that we had here in the department. And now we have moved ourselves to more advanced techniques in method-genomics, so that they are giving information that we could not even dream about six years ago. (Senior researcher, geosciences, 2015)

In epistemic terms the group, through a new research line, obtains the means to control a new property - the genetic makeup - of the epistemic object they work on: the lipid. This increases the number of properties the group can work. The high decomposability of control also means that the group leader can productively work with the researcher on this topic while she would not actually be able to carry out the genetics research herself. The senior researcher explains that this has been a process of learning for both her and the group leader:

*Interviewer:* Are you also now teaching [group leader] and [second professor] about genetics?

Yes, I think they learned a little bit. I mean, I learn from them the entire time about the lipids, but they also learn from me, so I think that is really nice. (…) because they have to read the papers I write. We have to discuss them together, so at the end they learn about it. The same way that I ended up learning about lipid techniques myself. (Senior researcher, geosciences, 2015)

Of course, for the group investing in this particular specialty comes with high strategic and technical uncertainty. It is not certain that the combination with genetics will ensure major new discoveries; it is not certain whether the group will be able to successfully use molecular genetics to find the pathways of their lipids.

The group leader did not only spend the Spinoza prize on this new researcher but used it also as a flexible reserve to be used as a “lubricant” in the research process, or to be able to react swiftly on new developments. She explains:

To give an example, we have [a visiting PhD-student]. She took more work on than she can handle. I already (…) predicted it would be too much, but alright, she is finding that out for herself now. So it would be good if she would stay a month longer at the institute. But, says [visiting PhD-student], I only have a scholarship for three months. So I ask, ‘well, how much do you get?’ That turned out to be 1200 Euros a month from which she pays everything. So I could say immediately, ‘then I will just pay 1200 Euros so you can stay a month longer,’ with the Spinoza prize that is just peanuts. (…) that is just an example of how that works as a kind of lubricant in our research machine. Another example, yesterday [senior researcher] came into my office with a question, ‘with a new PhD-student we want to do a new genetic research in [place].’ But that will be about 8000 Euros. Then I think, ‘well, we will see where we get it,’ I always know, ‘I have the money from my Spinoza.’ Or if I think, ‘I just want to do something completely different for a change and I don’t want to integrate it in a research proposal,’ then I can just hire a PhD-student or an analysis from the Spinoza money. (Professor, geosciences, 2015)

Remarkable in this case is that the use of the Spinoza prize has both an organizational and an epistemic dimension. The group leader has clear scientific ambitions to change her field by trying out a new combination of disciplines, or work with a visiting PhD-student on something that looks promising. To be actualized, such epistemic choices need organizational support. In the case of the new researcher, the management board had to approve funds for such a position and a new PhD-student or data collection for new genetic research needs to be written in project proposals. The Spinoza prize offers an organizational solution for these epistemic ambitions as it gives the group leader autonomy to make her own decisions as to what or whom to fund.

The use of the third Spinoza prize, in the mathematics group, also shows a combination of epistemic and organizational concerns. The professor explains that she wanted to recreate the environment in which she recently worked, before coming to the Netherlands. For that she needed someone who could build such an environment:

I came from [university], and there it was like heaven on earth. (…) So what I wanted to do with the Spinoza-award was to recreate as far as possible the research environment of [last university] here. (…) That money will attract new money, because the first flow funding is determined by formulas, and the more second flow funding you receive, the more first flow funding you get. So I could do all kinds of things with that and the most important thing I did, is that I took [professor] away from [other university]. And that is not only a good mathematician, but also really good in running things and knowing what needs to happen when you want to achieve certain things. (…) I believe that [she] in the end was paid for five or eight years from the Spinoza. So about half of the Spinoza-award went to [her], but that was more than an excellent investment. (Professor, mathematics, 2015)

The professor was able to bring in another professor who had a good eye for management and organizing an institute in a successful way, both as a community and regarding funding. This professor explained what the Spinoza winner wanted when he came in:

She wanted that [own institution] would radiate that same energy that you have in [famous institution], there all kinds of things happen without having to organize it and that was clearly not the case when she started here. Now it is. You can go to seminars all the time because we have so many PhD-students and postdocs that they organize things all the time. More than you can keep track of. That was totally different before. If something had to happen you had to organize such a seminar yourself and get people to come there. (Professor, mathematics, 2015)

The epistemic dimension of this choice was less geared towards novelty than in the previous case. The professor that came into the institute paid for by the Spinoza prize had worked on her dissertation with the Spinoza winner and collaborated with her in different institutes. Both professors work on a relatively similar subject in mathematics and they have published together, indicating a close collaboration. Together they were able to bring talented young researchers into the institute - PhD-students, postdocs and assistant professors. Both the professors and the younger staff became successful in terms of funding in the years after.

## Discussion and Conclusion

In this article we set out to understand the difference between epistemic and organizational properties of research projects funded by prizes versus project grants. We compared the organizational and epistemic properties of research funded from five project grants (ERC Advanced Grants and Vici grants) with those of three Spinoza prizes. We find that the organizational and epistemic properties of project proposals are prestructured by the standards of the research field and the assumed conservatism of peer review panels that judge project proposals.

For these peer review panels a particular model of scientific organization (Laudel and Gläser 2014: 1215) in which a principal investigator works with a team of PhD-students, postdocs and senior staff members is the standard. While the budget structure of the project grants is very open, the peer review process prestructures the organizational and epistemic characteristics of the project proposals.

The project grants contain high-risk elements, such as the ERC Advanced Grant regarding paleothermometers or the large-scale survey among a population that is difficult to reach, but these high-risk elements are not the core of the proposal. These elements can fail without endangering the project at large. In the interviews this particular way of coating a proposal with a risky element is explained as an effect of the project proposals going through the highly selective peer review processes. Although we have not analyzed these peer review processes in detail in our research, our interview material suggests that peer review panels do not seem inclined to fund studies that cannot already build on a sound basis. A professor in geosciences explains:

The truth is that a proposal does not always contain innovative research. If it would only contain innovative research, you would only propose things you are planning on doing and you do not know if these will work or not. That is really frontier research, but that will never make it because reviewers will always ask: ‘Yes, but how do you know if this will work?’ (Professor, geosciences, 2015)

Our analysis shows that the delegation of most of the research tasks to junior staff also influences the perceived degree of risk that can be taken. Both senior and early career researchers indicate that the career prospects of the particular researcher carrying out the work can strongly determine the degree to which the project deviates from common standards. For PhD-students it is important that in the end their research results in a dissertation. For postdoctoral researchers, the duration of their current contract in combination with the degree to which their current CV qualifies them for a new position or research grant, determines the degree to which they are willing to take risk in their research. If they expect to need an extra publication on the short term to get a new position or acquire a grant, they will tend to take less risks than when they have a contract which continues for several years, in combination with an impressive publication list.

The three Spinoza prize case studies diverge from project-funded research in three ways; A more flexible use, and adaptation of use of funds during the research process compared to project grants; investments in the larger organization which have effects beyond the research project itself; and, closely related, a deviation from epistemic and organizational standards. The two latter elements show the intertwined nature of the organization of an institute, group or research project and the epistemic properties of research done in an institute, group or research project.

First, the flexibility of the budget structure is important, as it allows for a more gradual use of the funding over a longer period of time. Two of the professors we interviewed, in humanities and mathematics, felt hindered by the way in which a project grant is organized as funds are distributed at once. The professor in the humanities group does not have a large number of PhD-students or postdocs and is not interested in using her funds, or acquiring additional project grants, to hire more staff. The extent to which the professor can make use of the work of PhD-students, the decomposition of control, is limited. As such, the professor does not believe that building a large group is an effective way of establishing and institutionalizing a research tradition. She argues:

I already have enough money. And I … received that million from [institute that awarded the second prize] on top of the Spinoza prize, this means that I now can devote 180% of my time to research. And I also think that there is an upper limit to what you can purposefully do with respect to increasing scale, to large-scale research in the humanities. (Professor, humanities, 2015)

In the mathematics case, the set time frame of projects is a problem especially in relation to the low degree of decomposability of control and the supervision relation. Supervising a PhD-student is, similar to the humanities case, seen as very laborious. This professor of mathematics who received a smaller Vidi grant (800,000 Euros) from the same funding scheme of which the Vici is part, explains:

Yes, yes. To put it less extremely: I would not have minded if my Vici project was to run for seven or eight instead of five years. I don’t know, maybe I would have still finished all the money in five or six years, but I would have had the feeling that I would have had the possibility to spread it a bit more.

*Interviewer:* Is that typical for Mathematics, you think?

I think so, maybe, at least typical and also not everywhere in Mathematics, but especially for those parts of Mathematics in which PhD supervision is so labor-intensive that you cannot realistically supervise more than two/three PhD-students at the same time. (Professor, mathematics, 2015)

Also in the geosciences case the Spinoza prize is used for a far longer period of time. The professor even explained that she still did not spend all of it, and did not plan on doing so immediately. Moreover, there is much less homogeneity in the ways in which the money is spent in the Spinoza prize cases. Where the project grants were spent on PhD-students and postdocs, the Spinoza prizes were spent on networks, student-assistants, tenure track staff members and all kinds of small projects. Both in the geosciences and the humanities case studies the professors use their prize to help young scholars to spend extra time in the lab or work on a project proposal without having to teach.

Second, in all three cases, the professors are using their Spinoza prize to intervene, first and foremost, in an organizational entity (what this entity is varies across cases). The aim of these interventions is to bring about epistemic innovation at the level of their own research but also at a larger scale. The Spinoza prize in the humanities is used to create a network of scholars across Europe, as well as a digital infrastructure and a small local organization of mostly student-assistants to organize the network as well as the digital infrastructure. In the geosciences group a new tenure track researcher is hired who can enrich the research agenda of the group through her knowledge of genetics. In the mathematics group a new professor is hired who is able to create a more lively and vibrant academic community. In all three cases, the focus is not on hiring PhD-students to execute preconfigured research projects but to establish something that goes beyond the individual researcher.

Third, at the same time, these organizational interventions have epistemic effects that are different from the research funded by the project grants. Especially in the humanities and geosciences case this becomes clear. The humanities professor has the ambition to understand a cultural process across a large variety of cultural communities and cultural expressions. These cultural communities and cultural expressions are separate research domains of all kinds of experts and she is unable to become an expert in everything. Therefore, in order to reach the goal, it is necessary to work in such a networked form. However, establishing such a networked form is, according to her, not feasible through a project grant. Indeed we observed that the data collection process, in which external experts are involved, evolved during the time we were in the group. The professor shifted focus towards specific types of cultural expressions (e.g., ranging from operas to statues) and towards specific cultural communities (e.g., building a stronger dataset on Scandinavian communities), and created workshops around these emerging themes during the research process. The ability to use funding in a flexible way affords the development of a comparison across time and cultural communities in a reflexive and iterative manner. Being able to do so is crucial to develop the empirical analysis for the theoretical argument the professor makes.

In both the geoscience and mathematics groups, the professor uses part of the Spinoza prize to fund a new tenure track position. In the geoscience group this position is outside of her own expertise, and explicitly entailed bringing novel insights and methods to the group. While the new researcher has become quickly embedded in the lab-structure of the group, this structure has not changed, she has been able to intervene in the epistemic direction of the group as a whole. It is hard to conceive how the group leader could arrive at this epistemic innovation, moving into genetics, through a project proposal: she does not have a track record in this field and could not supervise PhD-students on the topic.

What the last two points show is that prize funding enables researchers to deviate from epistemic and organizational standards. It is, however, more difficult to assess the amount of risk or strategic and technical uncertainty in comparison to project funded research. By avoiding the delegation of research tasks to PhD-students, senior researchers can certainly use the prize funded research to plan their research outside of the time-frame of a dissertation. Moreover, prize funding shares characteristics with recurrent block funding by offering ‘organizational slack’: it permits the researcher to develop innovative ideas without strict evaluative protocols. The group leader in geosciences identified this as giving him the opportunity to experiment with new ideas. It can also be used to bypass management in the hiring of new permanent staff members.

Our analysis shows that funding arrangements structure how individual researchers, research groups and institutes organize research, whether they can deviate from their own research trajectory and how they organize epistemic innovation. We observed significant differences between affordances of project grants versus prizes, even when looking at highly prestigious and generous project grants without any thematic restrictions and limited demands in terms of project deliverables. One can assume that the differences will be much larger for project grants of smaller size, with more restrictions and/or organizational demands.

We have used the prize funding arrangement as a means to highlight the affordances and constraints of project grants. Prizes are, however, not a suitable alternative to project funding arrangements. Prizes such as the Spinoza prize are only awarded to the top of established scholars in the Netherlands. Very often these scholars have, in the years before receiving a Spinoza prize, received one or more large project grants. For instance, among the four Spinoza prize winners of 2017 there were a total of three ERC Advanced Grants (one grantee received two ERC Advanced Grants), two Vici grants, as well as an ERC Starting Grant and ERC Consolidator Grant.

With this study we do not argue for more prizes as a solution to project funding. Rather we have identified the adverse effects of a science system in which project proposals are a critical component of the main funding mechanism for distributing resources. Our study shows that even the ‘winners’ of the science system are restricted by project funding arrangements and that there is a need for funding that is not tied to a specific project for epistemic and organizational innovation to take place.

## Footnotes

1 The evaluation of Vici proposals is based on the innovative character of the proposal (counts for 40%), the academic merits of the applicant (40%) and on knowledge valorization (20%). The ERC advanced grant does not specify percentages but claims its sole evaluation criterion is academic merit (or excellence) of both the applicant and the proposal.

2 The evaluation of nominated scholars for the Spinoza prize is based on internationally recognized top quality (70%), attraction to young researchers (20%) and knowledge valorisation (10%).
